# The Physical Health Conditions in People With Gambling Disorder

**DOI:** 10.1192/bjo.2025.10104

**Published:** 2025-06-20

**Authors:** Nicholas Botha, Samuel Chamberlain, Jon Grant, Konstantinos Ioannidis

**Affiliations:** ^1^University of Southampton, Southampton, United Kingdom; ^2^University of Chicago, Chicago, USA

## Abstract

**Aims:** Gambling Disorder is a mental health condition in which people experience impaired ability to control or stop gambling behaviours despite adverse consequences. It is associated with psychiatric co-morbidities and risk factors for physical health conditions. There is growing exploration into physical health conditions associated with gambling disorder and this study sought to further contribute towards understanding that association. The first aim was to describe rates of physical health conditions. The second aim was to explore potential associations between physical health conditions and individuals’ sociodemographic characteristics.

**Methods:** Dataset one comprised aggregated anonymised subject-level data from clinical trials conducted in participants with gambling disorder (n=423). Dataset two comprised aggregated anonymised patient data from the NHS Southern Gambling Service (n=352). Descriptive characteristics of people with versus without physical health co-morbidities were presented. Statistical tests were undertaken to compare those with versus without physical health co-morbidities, independent t-tests were utilised to compare continuous variables whereas Chi Square or Fisher’s Exact test were utilised when comparing categorical variables.

**Results:** In dataset one 42.9% reported one or more physical health condition, the most frequent reported physical health conditions were musculoskeletal, cardiovascular, and endocrine and metabolic conditions. People in dataset one with physical health condition(s) versus without had significantly older age. In dataset two 27.1% reported one or more physical health condition, with respiratory, musculoskeletal, and endocrine and metabolic being the most reported. The presence of physical health conditions, musculoskeletal conditions, and endocrine and metabolic conditions was associated with significantly older age and female sex.

**Conclusion:** Increased age in individuals with gambling disorder is a crucial sociodemographic factor regarding physical health morbidity. In dataset two being female was identified as a risk factor for having physical health morbidity. Implementation of treatments targeting these risk factors may reduce the public health and individual health burden of gambling disorder.

